# Therapeutic Efficacy of *Nyctanthes arbor-tristis* Flowers to Inhibit Proliferation of Acute and Chronic Primary Human Leukemia Cells, with Adipocyte Differentiation and in Silico Analysis of Interactions between Survivin Protein and Selected Secondary Metabolites

**DOI:** 10.3390/biom10020165

**Published:** 2020-01-21

**Authors:** Saumya Nishanga Heendeniya, Lakshika. Rangi Keerthirathna, Chamalika Kanthini Manawadu, Indeewarie Hemamali Dissanayake, Rizwan Ali, Abdullah Mashhour, Hajar Alzahrani, Pahan Godakumbura, Mohamed Boudjelal, Dinithi Champika Peiris

**Affiliations:** 1 Department of Biomedical Sciences, British College of Applied Studies, BCAS City Campus, Colombo 00600, Sri Lanka; saumyanh93@gmail.com; 2 Department of Zoology, Faculty of Applied Sciences (Center for Biotechnology), University of Sri Jayewardenepura, Nugegoda 10250, Sri Lanka; rangee9183@gmail.com (L.R.K.); chamalika.manawadu@yahoo.com (C.K.M.); indeewari92@gmail.com (I.H.D.); 3 Medical Research Core Facility and Platforms, King Abdullah International Medical Research Center, King Saud bin Abdulaziz University for Health Sciences, Ministry of National Guard Health Research, Riyadh 11481, Kingdom of Saudi Arabia; aliri@ngha.med.sa (R.A.); mashhoura@ngha.med.sa (A.M.); alzahraniha@ngha.med.sa (H.A.); boudjelalmo@ngha.med.sa (M.B.); 4 Department of Chemistry, Faculty of Applied Sciences (Center for Instrumentation Facility), University of Sri Jayewardenepura, Nugegoda 10250, Sri Lanka; pahanig@sjp.ac.lk

**Keywords:** *Nyctanthes abor-tristis*, human breast carcinoma, primary peripheral blood mononuclear cells, acute myeloid leukemia, chronic lymphocytic leukemia, obesity, hypoglycemia, molecular docking, survivin protein, gas chromatography/mass spectrometry

## Abstract

Although the antidiabetic efficacy of *Nyctanthes arbor-tristis* flowers has been reported, antiproliferative and anti-obesity activities are yet to be explored. We examined the anti-obesity and antiproliferative potentials of different fractions (hexane, chloroform, ethyl acetate, methanol) of *N. abor-tristis* flower extract for the first time using 3T3-L1 cells, primary peripheral blood mononuclear cells (PBMC) isolated from healthy and adult acute myeloid (AML) and chronic lymphocytic leukemia (CLL) patients, recombinant Jurkat T cells, and MCF7 cell lines. The in vitro hypoglycemic activity was evaluated using the inhibition of α-amylase enzyme and glucose uptake by yeast cells. The percentage glucose uptake and α-amylase inhibitory activity increased in a dose-dependent manner in the crude and the tested fractions (hexane and ethyl acetate). Inhibition of the 3T3-L1 cells’ differentiation was observed in the ethyl acetate and chloroform fractions, followed by the hexane fraction. Antiproliferative analyses revealed that *Nyctanthes* exerted a high specific activity against anti-AML and anti-CLL PBMC cells, especially by the hexane and ethyl acetate fractions. The gas chromatography/mass spectrometry analysis indicated the presence of 1-heptacosanol (hexane fraction), 1-octadecene (hexane and chloroform fractions), and other organic compounds. Molecular docking demonstrated that phenol,2,5-bis(1,1-dimethylethyl) and 4-hydroxypyridine 1-oxide compounds showed specificity toward survivin protein, indicating the feasibility of *N. abor-tristis* in developing new drug leads against leukemia.

## 1. Introduction

In the 21st century, noncommunicable diseases, such as cancer, have been categorized as one of the most prominent barriers to increasing life expectancy [1]. In 2018 alone, it was projected that there would be an additional 18.1 million cases of cancer worldwide [2]. The leading form of cancer is credited as being lung cancer, followed by female breast cancer, which is responsible for over 25% of all cancer-related incidents in women [3]. Breast cancer is presently being characterized as the leading cause of cancer-related deaths in over 100 countries. Countries occupying the continents of South America, Africa, and Asia have been observed as having increased incidences of breast cancer, which is being related to factors ranging from genetics to a combination of demographic, social, and economic factors [2]. Among the cancers, leukemia occurs mainly as a result of the abnormal multiplication of white blood cells and their precursors [4]. Leukemia is prevalent among both children and adults, occurring as a result of alterations in cell regulatory process causing uncontrolled proliferation of bone marrow hematopoietic stem cells. In the United States alone, about one hundred thousand new cases are reported each year. [5]. Though the prevalence of leukemia is higher in white males, high incidences of leukemia can also be found among children in Asian countries such as Sri Lanka [6]. Leukemia can be divided into acute and chronic forms, where in acute leukemia, the abnormal blood cells remain immature and fail to carry normal functions. In contrast in chronic leukemia, the abnormal cells may continue to function normally. Further, depending on affected blood cells, leukemia can be categorized into lymphoblastic and myeloid leukemias. With lymphoblastic and myeloid leukemias, the malignant changes occur respectively in precursor cells of lymphocytes and red blood cells, and other white blood cell types and platelets. Hence, on the basis of the above categories, leukemia can be divided into four subtypes: acute and chronic lymphoblastic leukemias (ALL, CLL), and acute and chronic myeloid leukemias (AML, CML) [7]. Among the four subtypes, the acute myeloid leukemia is considered as the most common type on the basis of its highest incidences in both new cases and deaths in leukemia [8]. 

Similarly, obesity and diabetes are the most commonly related metabolic disorders in the world [9]. Both diabetes and obesity are increasing all over the world, including in South Asian countries such as Sri Lanka [10]. Rapid urbanization associated with lifestyle changes has led to an increase in the consumption of refined carbohydrates; this results in a rapid increase in blood glucose levels and can lead to diabetes. High blood glucose levels are linked with obesity, as excess blood glucose is converted into adipocyte tissues [11]. The inhibition of carbohydrate digestive enzymes, including α-amylase, is a potential treatment against both diabetes and obesity [12]. The inhibition of cells maturing into adipocyte tissues will be a promising therapeutic agent against obesity. 

Conventional medical treatments against these noncommunicable diseases are notorious for their short-term and long-term side effects. Anticancer treatments, such as chemotherapy, pose side effects that lead to tremendous patient discomfort, varying from nausea; hair loss; and gastrointestinal, reproductive, and neural disorders. These factors are a concern for both clinicians and patients [13]. There is similar concern for antidiabetic medicine, such as metformin, pioglitazone, and anti-obesity drugs. These drugs are reputed as causing lactic acidosis, weight loss, hypoglycemia, and the induction of nausea [14]. 

Organic plant-based remedies are popular among Ayurveda and other modes of traditional medicine, as they are free of the harmful biproducts of conventional medicine [15]. At present, the focus on plants with a high polyphenolic content has increased due to their documented chemoprotective, antidiabetic, and anti-obesity potential [16], as well as the selective cell-killing ability [17] of such plants. 

*Nyctanthes arbor-tristis* (family—Oleaceae), which is commonly referred to as night jasmine or sepalika, is a popular medicine that has been prescribed by traditional practitioners in Sri Lanka for generations. The plant is used as a remedial solution for a number of disease conditions due to its broad spectrum of biological activity [18]. Analysis of the plant’s leaves, fruits, and seeds has revealed the presence of a number of phytochemicals vital in the treatment of ailments. Flower extracts of night jasmine have been shown promising antibacterial, antiviral, antifilarial, and antioxidant activities with minimum side effects compared to common among conventional medicines [19].

It has been documented that *Nyctanthes arbor-tristis* possess antidiabetic and anticancer potentials, and it is also used as an expectorant, hair tonic, stomachic, carminative, and astringent [20]. Previously, we have shown that a hot infusion of *N. arbor-tristis* flower was capable of reducing blood glucose levels in mice at 3 h post-treatment [21]. However, its antiproliferative potentials are yet to be explored. 

Survivin is an inhibitor of the apoptotic protein family, inhibiting caspase-9 to prevent the initiation of the mitochondrial pathway of apoptosis [22]. The expression of survivin in normal tissues is tightly regulated and plays a pivotal role in the regulation of apoptosis in leukemic cancer cells [23]. Hence, it has become an important therapeutic target for cancer management. In silico, protein–ligand docking has become a vital tool for drug discovery. In virtual screening it is important that the ligands are selected with a natural conformation that can be efficiently bound with a protein of interest. Molecular docking is capable of accurately predicting active sites and the ligand structure, leading to drug design [24]. 

The present study aimed to identify the compounds responsible for antidiabetic potential, and we also investigated the adipocyte differentiation and anticancer potentials against human primary breast cancer cells and the NIH-3T3 L1 cell line. We believe that this study also provides insight on the compounds in *N. arbor-tristis* that promote anticancer and anti-obesity activities and on the formation of the ligand–protein complex. 

## 2. Materials and Methods

### 2.1. Chemicals and Equipment

The main pieces of equipment used in the study were a gas chromatography/mass spectrometry (GC/MS; Spectra lab Agilent-7890, Markham, ON, Canada) instrument, a rotary evaporator (Buchi rotovapor, R-124 digital, New Castle, DE, USA), and a UV-VIS spectrophotometer (Genesys 10S UV, Vis, St Louis, MO, USA). All chemicals and reagents were of analytical grade and obtained from Sigma-Aldrich, Taufkirchen, Germany. Water, when used, was distilled using distillation apparatus. All culture media and standard antibiotic disks were purchased from Sigma Chemical Company Ltd (St. Louis, MO, USA).

### 2.2. Plant Material

Fresh flowers were collected early in the morning (06:00–07:00) from Colombo, Sri Lanka (6.9271° N, 79.8612° E) and were authenticated by Prof. B. M. P. Singhakumara at the Department of Forestry and Environmental Science, University of Sri Jayewardenepura. 

### 2.3. Preparation of the Aqueous Extract and Its Fractions

The flowers were thoroughly washed with distilled water and air dried in the dark at room temperature. The flowers were powdered using a mechanical blender. The extraction procedure was conducted according to the method of Lakmal et al., [25] with modifications. The powdered flowers (15 g) were mixed with 100 mL of distilled water and subjected to sonication at 25 °C for three 90 min periods. The extract was filtered through Whatman filter paper no. 1, freeze-dried, and stored at −20 °C until further use.

The freeze-dried crude extract was subjected to sequential solvent–solvent fraction with different organic solvents, hexane, chloroform, ethyl acetate, and methanol. The obtained fractions and crude aqueous extract were filtered through paper filter Whatman no. 1 to remove the solid particles and then concentrated using a rotary evaporator (BUCHI, Rotavapor, R-300, New Castle, DE, USA) and used to conduct the assays.

### 2.4. Total Phenolic Content

The standard Folin–Ciocalteau method was used for the determination of the total phenolic content, according to the method described by [26]. A 100 µL aliquot of dried residue of the fractions, crude extract dissolved in deionized water (1 mg/mL), or a standard solution of gallic acid dissolved in deionized water (0.1, 0.05, 0.025, 0.0125, and 0.003125 μg/mL) was added to a test tube in distilled water, and 100 µL of each concentration was added to an equal volume of tenfold diluted Folin–Ciocalteau reagent followed by 2% Na_2_CO_3_ in a test tube. The mixture was incubated for 2 h at room temperature in the dark, and the absorbance was measured at 760 nm in an UV visible spectrophotometer against a blank solution. The procedure was repeated for both methanol and aqueous extracts at a concentration of 1 mg/mL. The total phenolic content was expressed in milligram equivalents of gallic acid (GAE) per gram of extract. 

### 2.5. Total Flavonoid Content

The total flavonoid content was measured by the aluminum chloride colorimetric assay [27]. A 100 µL aliquot of dried residue of each fraction, crude extract (1 mg/mL), or a standard solution of quercetin dissolved in distilled water (20, 40, 60, 80, and 100 μg/mL) was added to test tubes. To the test tube, 5% NaNO_2_ was added, and after 5 min 10% AlCl_3_ was added. After 5 min, 200 µL of 1M NaOH was added, and the volume was filled up to 1 mL with distilled water. The solution was mixed, and the absorbance was measured against the blank at 510 nm. The total flavonoid was expressed as milligram of quercetin equivalents per gram of dry plant.

### 2.6. Gas Chromatography/Mass Spectrometry Analysis

Only the active fractions were analyzed by a gas chromatography/mass spectrometry (GC/MS) instrument equipped with a triple axis detector and a Hewlett Packard 7683 B series injector, with a mass spectrometer transfer line temperature of 250 °C. The gas chromatograph (GC) was equipped with a fused silica capillary column (HP-5MS; 30 × 0.25 mm), with a film thickness of 1.0 μm. The oven temperature was held at 50 °C for 5 min and raised from 50 to 250 °C at a rate of 2 °C/min, employing helium gas (99.999%) as a carrier gas at a constant flow rate of 22 cm/s. One milliliter of the sample (1 mg dissolved in 1 mL of methanol) was injected into the column using the split mode (split ratio: 1:30). The GC/MS analysis was carried out on an Agilent Technology Network mass spectrometer (model 5975 series) coupled to a Hewlett Packard gas chromatograph (model 7890 series) equipped with the NIST08 library software database. Mass spectra were taken at 70 eV. The scanning range was 1–550 m/z, and the injector temperature was set at 200 °C. The compounds were screened using the database of the NIST08 library. The mass spectrum of an individual unknown compound was compared with the known compounds stored in the software database library.

### 2.7. Alpha-Amylase Inhibitory Assay

The dosage was selected according to previous studies done by [21], where their middle dose (500 mg/kg) was equivalent to the quantity prescribed by traditional practitioners. We back-calculated the doses and selected 1.5, 3, and 5 mg/mL for each fraction. 

Starch solution (0.5% *w*/*v*) was prepared by stirring soluble starch (0.25 g) in 20 mM sodium phosphate buffer with 6.7 mM NaCl (pH 6.9; 25 mL) in a boiling water bath for 15 min. Subsequently, 200 µL of the different concentrations (1.5, 3, and 5 mg/mL) of different fractions and acarbose (positive control) were mixed with 200 µL of the enzyme solution in a test tube and incubated at 25 °C for 30 min. Subsequently, the starch solution was added and incubated at 25 °C for 3 min. Finally, 200 µL of 3,5-dinitrosalicylic acid (DNS) color reagent was added to terminate the reaction, and then the solutions were placed in a water bath for 15 min. The final volume was made up with distilled water, and the absorbance was measured at 540 nm using a spectrophotometer. The 50% inhibitory concentration or IC_50_ value was calculated.

### 2.8. Determination of Glucose Uptake Capacity by Yeast Cells

Commercial baker’s yeast was washed with distilled water until clear supernatant fluids were obtained, and a 10% (*v/v*) of the suspension was prepared in distilled water. The different concentrations (1.5, 3, and 5 mg/mL) of the crude extract, the fractions, and the acarbose (positive control), with volumes of 300 µL, were mixed with 300 µL of 10 mM of glucose solution and incubated for 10 min at 37 °C. The reaction was initiated by adding 30 µL of yeast suspension and incubated at 37 °C for 60 min. After 60 min, the tubes were centrifuged (2500 rpm for 5 min), and the amount of glucose was estimated in the supernatant using an UV spectrophotometer [28]. The percentage inhibition of glucose uptake by yeast cells was calculated using the following formula:

% glucose uptake = (absorbance _control_ − absorbance _sample_)/absorbance _sample_ × 100.

### 2.9. Differentiation of 3T3 Cells into Adipocytes

The 3T3L1 cell line from mice is a continuous strain of 3T3 developed through clonal isolation. Cells can be induced to become adipose-like cells using a cell differentiation cocktail. The sub-confluent cultures (70–80%) were passaged, and thus cultures were never allowed to become fully confluent. Seeding of 2–4 × 10,000 cells per well in a 24-well cell culture plate was done in advance, with Dulbecco’s modified Eagle’s medium (DMEM) supplemented with 10% calf serum, 2 mM glutamine (GIBCO, Fisher Scientific UK Ltd, Loughborough, LE, UK), 50 units/mL penicillin (GIBCO), and 50 μg/mL streptomycin (GIBCO), and incubated at 37 °C in a humidified 5% CO_2_ atmosphere. 

To stimulate differentiation into adipocytes, the cells were grown in the mentioned media to confluence. Two days after, confluency differentiation was induced by adding 0.5 mM 3-isobutyl-1-methylxanthine (IBMX), 0.25 μM dexamethasone, and 1 μg/mL insulin in DMEM with 10% fetal bovine serum (FBS). After 2 days, the IBMX was removed along with the dexamethasone, but insulin was maintained for another 2 days. On the fourth day after inducing differentiation, the cells were cultured in DMEM with 10% FBS. The medium was changed every two days. Differentiation took 2 to 5 weeks. Differentiation began in patches, but with time a significant percentage of the population changed [29]. 

Triglycerides in cells were estimated using a commercially available triglyceride kit (Agappe Diagnostics Ltd. Kochi Kerala, India) according to the manufacturer’s instructions. The cells were washed with phosphate buffer saline (PBS), scraped, and lysed in homogenizing buffer (42 mM KCl, 1 mM EDTA, (ethylenediaminetetraacetic acid) and 50 mM tris pH 7.4), and the cell lysate was centrifuged at 3000× *g* for 10 min at 4 °C. The supernatant was assessed for triglyceride content. Similarly, the total protein in the supernatant was measured by a commercially available kit after 48 h of adipocyte differentiation with and without plant extracts [30].

### 2.10. Antiproliferative Activities

#### 2.10.1. Primary Carcinoma Cell and Ethical Approval

Cancer primary cells were obtained from patients in King Abdullah International Medical Research Center (KAIMRC) after signing approved consent. The study was approved by the Institutional Review Board (IRB) Ministry of National Guard Health Affairs, Kingdom of Saudi Arabia (RC13/267). Primary peripheral blood mononuclear cells (PBMC) were obtained from healthy, adult acute myeloid leukemia (AML), and chronic lymphocytic leukemia (CLL) patients. The human T lymphocyte cell line derived from acute T cell leukemia (Jurkat cells) and the human breast cancer cell line (MCF7) were obtained from the American Type Culture Collection (ATCC). 

#### 2.10.2. Cell Culture

The human breast cancer epithelial cell line MCF7 (HTB-22) was maintained in complete DMEM, whereas the human T lymphocyte cell line derived from acute T cell leukemia (Jurkat) (ATCC TIB-152) was maintained in complete RPMI-1640 media. The media was supplemented with 10% FBS, 50 units/mL penicillin, 50 ug/mL streptomycin (GIBCO), 2 mM glutamine (GIBCO), and 4.5 g/L glucose. The cells were cultured at 37 °C in a humidified 5% CO_2_ atmosphere. 

#### 2.10.3. CellTiter-Glo Assay

Cells were seeded at a predetermined density on a white 96-well plate and treated with drugs at different concentrations and incubated for 24 h. Cells were lysed with CellTiter-Glo luminescent cell viability assay reagent (Promega, Durham, NC, USA) for 15 min in the dark, and the luminescence was read using the Envision plate reader (Perkin Elmer, Waltham, MA, USA). Percentage cell growth was calculated relative to the DMSO (dimethyl sulfoxide)-treated cells [31].

### 2.11. Molecular Docking

#### 2.11.1. Protein Preparation

To analyze the anticancer effect of the active compounds, survivin protein (PBD ID: 1XOX) was retrieved from the protein databank. The protein was prepared for docking using the BioLuminate protein preparation wizard. The protein was optimized, and an energy minimization was conducted using the OPLS3 force field.

#### 2.11.2. Natural Compound Selection

From the tested fractions, compounds with recorded anticancer activities were selected for virtual screening. In the hexane fraction, an alkene, 1-octadecene (PubChem CID:8217), and a fatty alcohol, 1-hepatacosanol (PubChem CID: 74822), were selected, whereas from the chloroform fraction, phenol,2,5-bis(1,1-dimethylethyl) (PubChem CID: 79983), which is a phenolic compound, and 4-hydroxypyridine 1-oxide (PubChem CID: 23321), a pyridine derivative, were chosen. 

#### 2.11.3. Active Site Prediction

Due to the large size of the macromolecule, it was difficult to conduct a singular docking analysis of the entire protein. Therefore, all potential active sites were firstly mapped. The SiteMap software (Schrödinger, New York, NY, USA) analysis revealed two such potential active site regions on the protein. Grid preparation was conducted for each of these potential active sites.

#### 2.11.4. Ligand Preparation

The 3D structure of 1-octadecene, phenol,2,5-bis(1,1-dimethylethyl), and 4-hydroxypyridine 1-oxide molecules were obtained through PubChem. There was no 3D structure for 1-hepatacosanol, as the PubChem conformer generation has disallowed its generation because the molecule is too flexible. The ligands were prepared using LigPrep, (Schrödinger, New York, NY, USA) the OPLS3 force field was also applied to the ligands. 

#### 2.11.5. Virtual Screening

The docking analysis was conducted using the Schrödinger Glide docking software (New York, NY, USA). Flexible ligand docking was utilized, with the assumption that the protein displayed a rigid body structure. For each active site, the ligand pose with the most feasible glide docking score was selected. 

### 2.12. Statistical Analyses

Statistical comparison was carried out using Minitab 16 2.4.0 (Minitab Inc, State College, PA, USA). The results of the experiments were expressed as the mean ± standard deviation (mean ± SD) or mean ± standard error (Mean ± SE). One-way ANOVA was used to determine the significant differences between the aqueous extract and the fractions. If a significant difference were found, the mean values were compared using the two-sample *t*-test. The Pearson’s correlation coefficient was used for the correlation analysis. The *p*-value was set at *p* < 0.05. 

## 3. Results

### 3.1. Total Phenolic and Flavonoid Contents

The total phenolic contents are presented in Table 1. The total phenolic content in the hexane and chloroform fractions varied between 351 + 0.0003 and 180 ± 0.0003 mg GAE/g, respectively. However, the methanol fraction (450 ± 0.014 mg GAE/g) exhibited twice the phenolic amount of the ethyl acetate fraction (285 ± 0.001 mg GAE/g). However, the crude aqueous extract exhibited the highest phenolic content of 160 ± 0.06 mg GAE/g. Similar to the phenolic activity, the crude extract exhibited the highest total flavonoid content (600 ± 1.25 mg Quercetin (QE)/g), and the flavonoid content in the methanol fraction was more or less equivalent to the crude aqueous extract (528 ± 2.01 mg QE/g). Among the rest of the fractions, the order of the total flavonoid content was chloroform fraction (120 ± 0.93 mg QE/g) > ethyl acetate fraction (30 ± 0.03 mg QE/g) > hexane fraction (24 ± 0.06 mg QE/g).

### 3.2. Gas Chromatography/Mass Spectrometry (GC/MS) Analysis of Solvent Fractions

**Figure 1:**
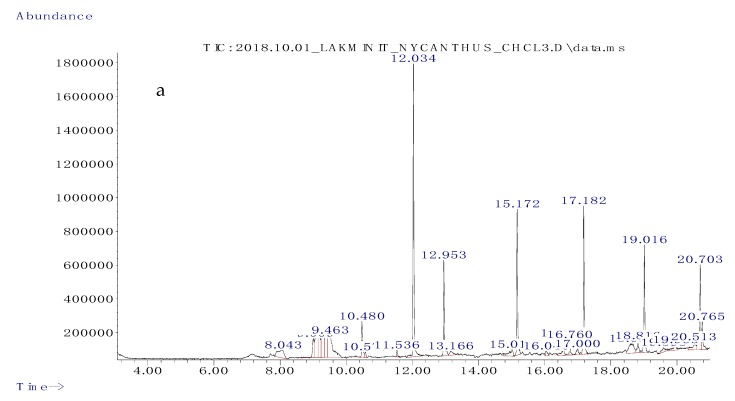

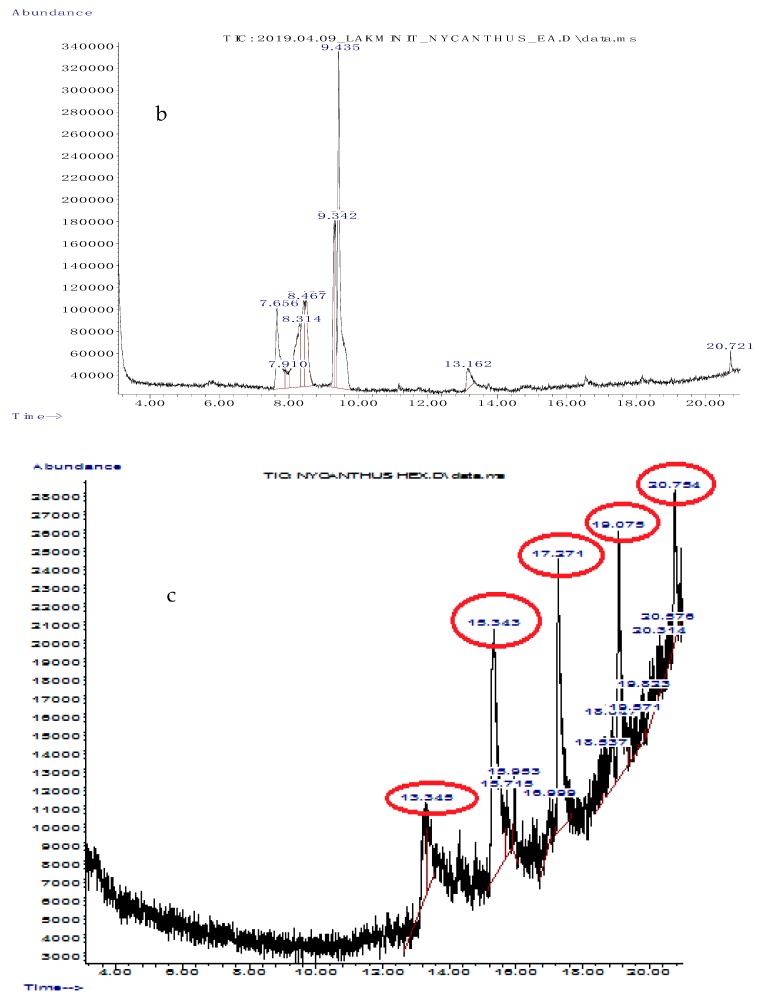
Chromatograms obtained from the gas chromatography/mass spectrometry (GC/MS) analysis of the chloroform fraction (**a**), ethyl acetate fraction (**b**), and hexane fraction (**c**) of the aqueous extract of *Nyctanthes arbor-tristis* flowers.

The chloroform fraction, ethyl acetate fraction, and the hexane fraction, which showed promising biological activities, were subjected to gas chromatography/mass spectrometry (GC/MS) analysis. On the basis of the NIST08 database, the mass-to-charge ratios obtained in the GC/MS chromatogram led to the identification of the chemical constituents present in the chloroform, hexane, and ethyl acetate fractions of aqueous *N. arbor-tristis* extract. The major peaks obtained in the gas chromatogram are depicted in Figure 1, including the chloroform fraction (Figure 1a), the ethyl acetate fraction (Figure 1b), and the hexane fraction (Figure 1c). 

A total of 10 main compounds were identified in the chloroform fraction (Table 2). These compounds were 4-hydroxypyridine-1-oxide, 2-aminopyrimidine-1-oxide, 2-thiophenecarbonyl chloride, 5-ethylcyclopent-1-ene-1-carboxylic acid, phenol,2,5-bis(1,1-dimethylethyl), 7-hexadecene, 1-octadecene, 1-eicosene, hentriacontane, and 1-docosene. Out of the 10 compounds, 9 had reported biological activity.

On the basis of the NIST08 database, eight compounds were identified in the ethyl acetate fraction of *N. abor-tristis*. As listed in Table 3, these compounds were cyclopentanecarboxamide, *N*-(2-fluorophenyl), 2-thiophenecarboxylic acid-3,5-dimethylcyclohexyl ester, 2,5-dimethylhexane-2,5-dihydroperoxide, adipic acid-ethyl propargyl ester, succinic acid (3,5-dimethyl cyclohexyl) ester, diethyl phthalate, and hexanedioic acid bis (2-ethylhexyl) ester. Out of the eight compounds, only three exhibited reported biological activities.

The compounds identified in the hexane fraction are listed in Table 4. Five main compounds were identified in the hexane fraction of the aqueous extract of *N. abor-tristis*. Among the five compounds, three showed recorded biological activities, namely, 2-chloropropionic acid, octadecyl ester, 1-octadecene, and 1-heptacosanol.

### 3.3. Alpha-Amylase Activity

The IC_50_ values for the inhibition of α-amylase activity are represented in Table 5. A significant (*p* < 0.01) inhibition of α-amylase enzyme was revealed among the hexane and ethyl acetate fractions of *N. abor-tristis*. The order of the potency of the α-amylase inhibition activity of the crude aqueous extract and the four fractions was hexane fraction (IC_50_: 0.665 ± 0.01) > ethyl acetate fraction (IC_50_: 0.718 ± 0.01) > methanol fraction (IC_50_: 1.504 ± 0.34) > crude aqueous extract (IC_50_: 2.223 ± 0.02) > chloroform fraction (IC_50_: 12.68 ± 0.09). The inhibitory activities exhibited by the hexane and the ethyl acetate fractions were respectively 73% and 71% more potent than the standard acarbose drug (IC_50_: 2.45 ± 0.23), and these fractions exhibited more or less equal inhibitory activities. 

### 3.4. Glucose Uptake by Yeast Cells

**Figure 2:**
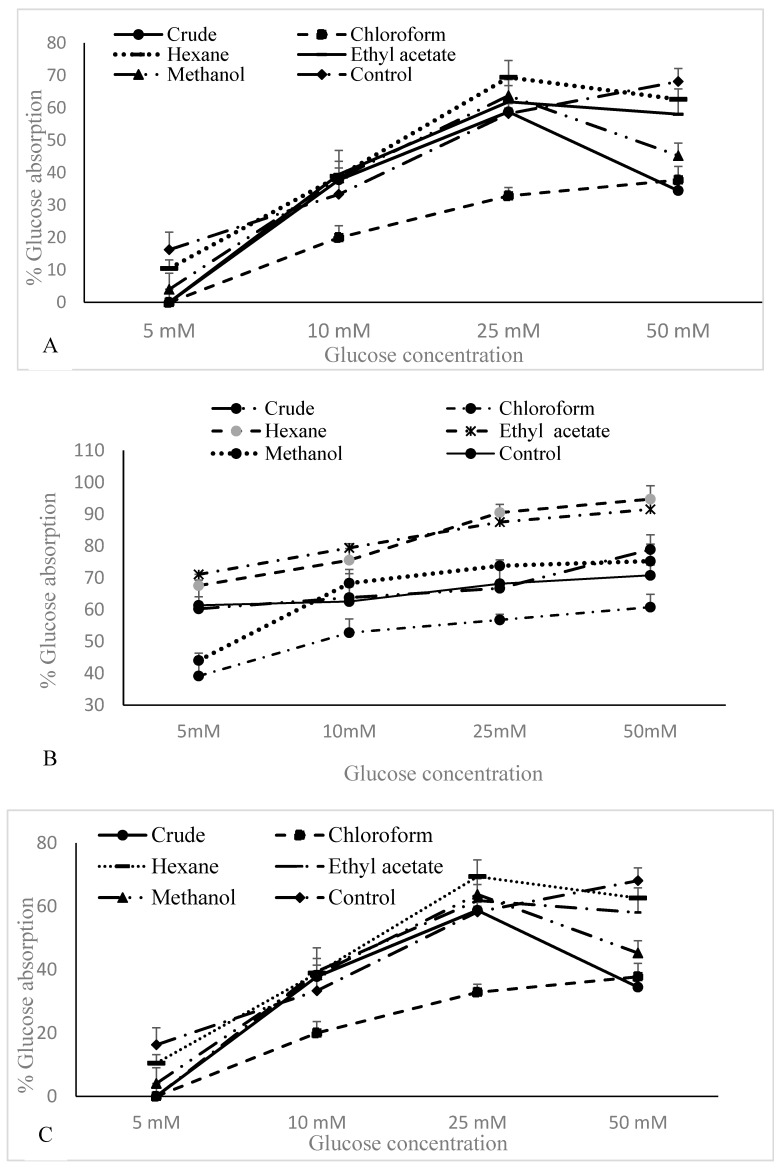
Percentage glucose absorption of crude aqueous extract of *N. abor-tristis* flowers, the fractions, and acarbose (control) at 1.5 mg/mL (**A**), 3 mg/mL (**B**), and 5 mg/mL (**C**) with different concentrations of glucose. Values are the mean ± SD of triplicate determination.

The glucose absorption capacity of the crude aqueous extract and the fractions (at different extract/fraction concentrations) in different glucose concentrations (5–50 mM) are depicted in Figure 2. The amount of glucose remaining in the medium after a given time served as an indicator of the percentage absorbed by the yeast cells. According to the results, the rate of glucose uptake in the yeast cells promoted a significant (*p* < 0.05) dose-dependent trend (*r* = 0.912), with the aqueous extract at 5 mg/mL dose for the other tested fractions, and the fraction following the same pattern. However, the linear increase of adsorption was observed only at 3 and 5 mg/mL glucose concentrations. At the lowest concentration of glucose, the crude extract and the fractions did not exhibit a significant linear relationship. However, at a 1.5 mg/mL dose, the standard drug, acarbose, and chloroform fraction exhibited a dose-dependent relationship (Figure 2A). At a 1.5 mg/mL dose, the glucose adsorption decreased at 50 mM of glucose concentration. As shown in Figure 2B,C, among the tested fractions, the maximum glucose absorption was observed with the ethyl acetate fraction (3 mg/mL: 91.5%; 5 mg/mL: 96.6%) and the hexane fraction (3 mg/mL: 94%; 5 mg/mL: 86%), compared to the standard acarbose (3 mg/mL: 71%; 5 mg/mL: 75%). At 5 mg/mL, the ethyl acetate fraction exhibited the highest glucose absorption of 96.6% at 50 mM of glucose concentration. In the presence of 5 mM glucose, zero absorption was recorded at 1.5 mg/mL doses of the crude extract, the chloroform fraction, and the ethyl acetate fraction.

### 3.5. Differentiation of 3T3 Cells 

**Figure 3:**
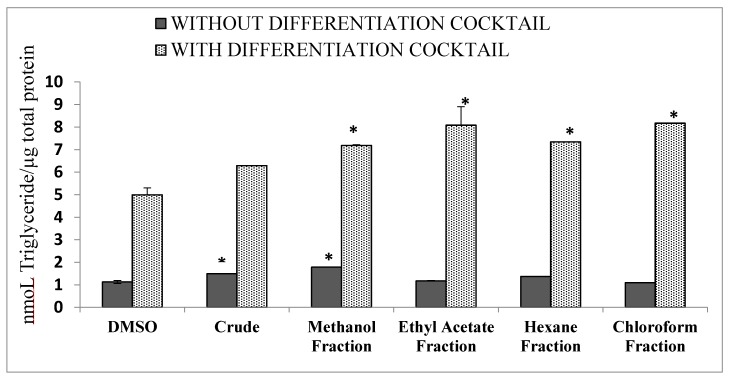
Effect of the crude aqueous extract and four fractions on 3T3L1 adipocyte differentiation with and without differentiation media. The quantification of intracellular triglycerides content and the total protein content is shown. Data were obtained from three independent experiments. The absorbance value is given as the mean ± SD. * *p* < 0.05. DMSO: dimethyl sulfoxide.

The crude aqueous extract and its fractions (chloroform, hexane, ethyl acetate, and methanol) were assessed on the differentiation of pre-adipocyte 3T3L1 cells into adipocytes with and without the IBMX differentiation cocktail, and lipid accumulation was determined. Compared to other fractions, the treatment with the ethyl acetate fraction and the chloroform fraction reduced lipid accumulation in the absence of the differentiation cocktail, which was more or less equal to the standard DMSO alone (crude aqueous extract: 20%; methanol fraction: 40%; hexane fraction: 44%; ethyl acetate and chloroform fractions: 60%). The ethyl acetate, hexane, and chloroform did not increase the differentiation in the absence of the differentiation cocktail (Figure 3). 

### 3.6. Antiproliferative Activity

The order of the potency of proliferation inhibition of the crude aqueous extract and the four fractions recorded in PBMCs (prepared from CLL patients) was ethyl acetate fraction (IC_50:_ 60.3 µg/mL) > crude aqueous extract (IC_50_: 90.1 µg/mL) > chloroform fraction (IC_50_: 98.6 µg/mL) > methanol fraction (IC_50_: 138 µg/mL) > hexane fraction (IC_50_: 273 µg/mL). The control, doxoroubicin, exhibited an IC_50_ value of 6.08 × 10^−2^ µg/mL, as depicted in Table 6. 

The crude aqueous extract exhibited the highest inhibitory activity against PBMC from acute myeloid patients (IC_50_: 1262 µg/mL) and PBMC from healthy individuals (IC_50_: 1588 µg/mL). The methanol fraction showed the highest inhibitory activity against Jurkat cells (IC_50_: 733 µg/mL), followed by the crude aqueous extract (IC_50_: 987 µg/mL) and the hexane fraction (IC_50_: 1174 µg/mL). The order of potency of the inhibitory activities in the MCF7 breast cancer cell line was hexane fraction (IC_50_: 717.6 µg/mL) > methanol fraction (IC_50_: 1016 µg/mL) > ethyl acetate fraction (IC_50_: 1493 µg/mL) > crude aqueous extract (IC_50_: 1664 µg/mL) > chloroform fraction (IC_50_: 38,210 µg/mL). However, the IC_50_ values exhibited by the crude extract and the fractions against AML and CLL (except for the hexane fraction against AML patients) was lower than the control cell line. In general, the chloroform fraction exhibited the least activity against all the primary cell lines used in the study. 

Bartmanska [47] introduced the selectivity index (SI) to determine the specificity of a drug against respective cancer cells. This can be calculated as a ratio of IC_50_ for a normal cell line and the respective cancerous cell line (SI = IC_50_ for normal cell line/IC_50_ for cancerous cell line). According to Bartmanska [47], if the SI values are >1.0, this indicates that the compound possesses considerable anticancer specificity, whereas a SI much greater than 1.0 indicates high selectivity. Table 7 displays the selective index for PBMC-AML and PBMC-CLL cells.

The SI values >1.00 indicate that a compound or a drug exhibits significant anticancer specificity, and if the SI value is much larger, then a compound/drug is highly selective. All investigated extract/fractions exhibited high selectivity toward the PBMC-CLL cells (SI_B_ > 1.00). The chloroform and ethyl acetate fractions exhibited very high selectivity against the PBMC-CLL cells (SI_B_ = 98.20 and 102.5, respectively), followed by the methanol fraction (SI_B_ = 48.22). The selectivity exhibited by the standard drug against PBMC-CLL cells (SI_B_ = 1.5) was much less compared to the fractions used in the study. The highest selectivity to the PBMC-AML cells (SI_A_ = 3.35) was exhibited by the methanol fraction followed by the ethyl acetate fraction (SI_A_ = 2.06). The hexane fraction and the standard drug did not exhibit any selectivity against the PBMC-AML cells (SI_A_ = 0.03).

### 3.7. Molecular Docking 

**Figure 4:**
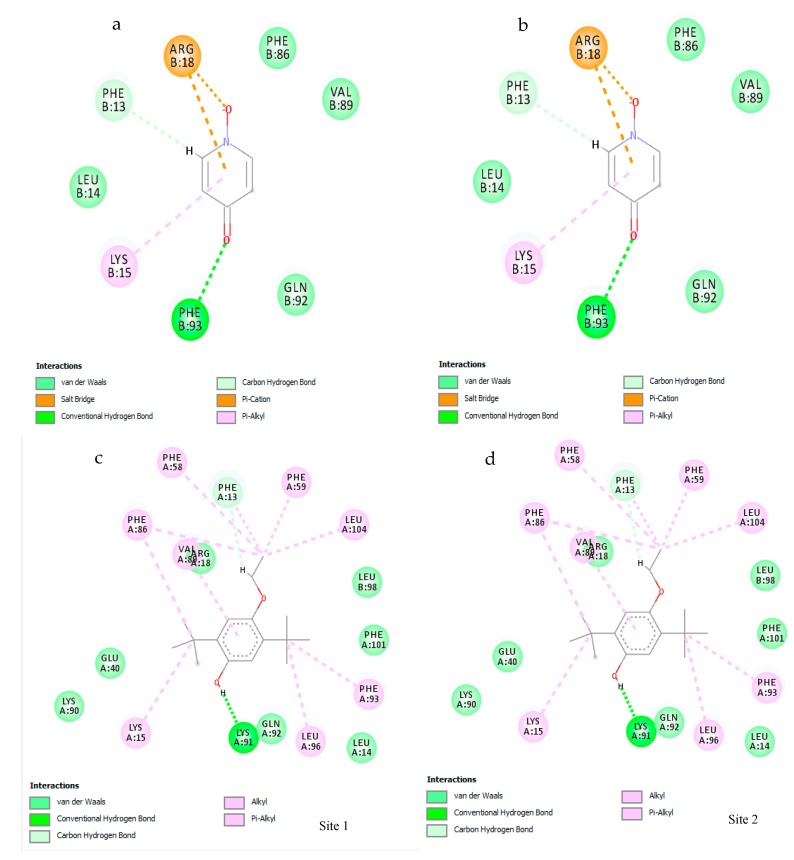
Two-dimensional interaction diagram for the protein–ligand complex. Interactions of the five active sites situated within the survivin protein and the phenol,2,5-bis (1,1-dimethylethyl) ligand (**a**,**b**) and with survivin and the 4-hydroxypyridine 1-oxide ligand (**c**,**d**).

SiteMap software analysis revealed that, among the tested secondary metabolites, only two ligands—phenol,2,5-bis(1,1-dimethylethyl) (PubChem CID: 79983) and 4-hydroxypyridine 1-oxide (PubChem CID: 23321)—displayed interactions with survivin protein. Analysis of both phenol,2,5-bis(1,1-dimethylethyl) (Figure 4a,b) and 4-hydroxypyridine 1-oxide (Figure 4c,d) showed two active sites, which formed a complex with the survivin protein molecule.

**Figure 5:**
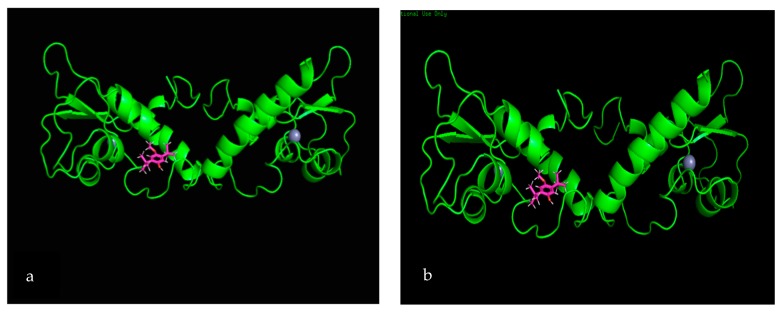
The three-dimensional surface structure forming a complex between the survivin protein–ligand phenol,2,5-bis(1,1-dimethylethyl) active site 1 (**a**) and active site 2 (**b**). It clearly shows the ligand (pink) occupying the active sites’ gorge via van der Waals forces (light purple).

Both active sites of the phenol,2,5-bis (1,1-dimethylethyl) ligand showed an approximately similar glide docking score of −5.449 kcal/moL and −5.269 kcal/moL. Two-dimensional analysis of the active site showed the involvement of one strong hydrogen bond with Lys91 residue and a number of van der Waals forces between the ligand and the residues from the protein (Figure 5a,b). 

**Figure 6:**
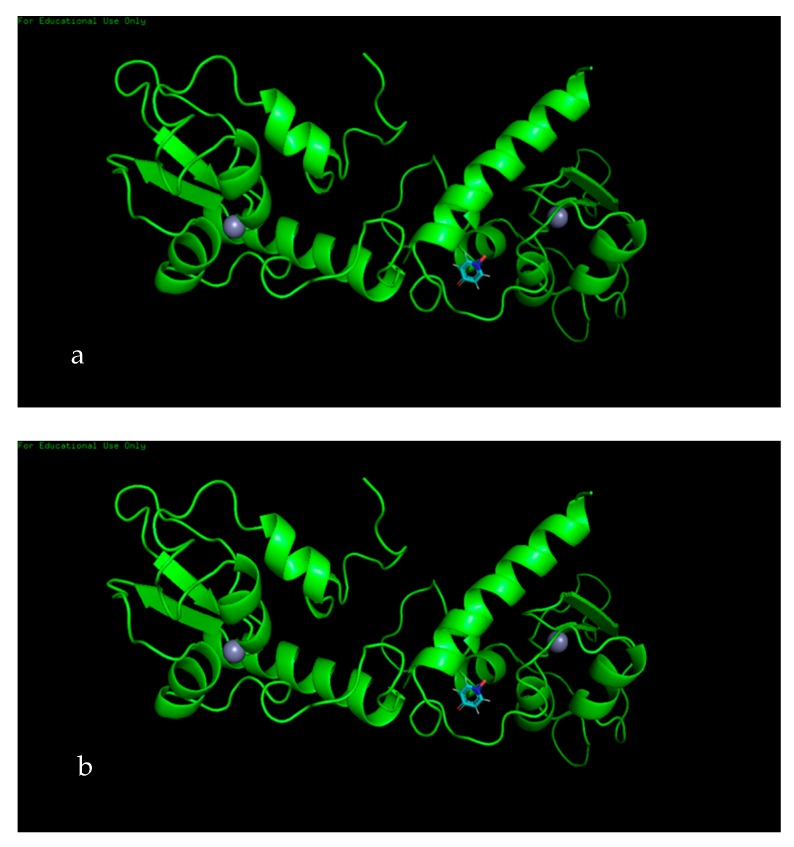
The three-dimensional surface structure forming a complex between survivin protein and ligand 4-hydroxypyridine 1-oxide active site 1 (**a**) and active site 2 (**b**). It clearly shows the ligand (blue) occupying the active sites’ gorge via van der Waals forces (red).

The 4-hydroxypyridine 1-oxide (PubChem CID: 23321) ligand displayed two active sites with the survivin protein molecule, with a glide score of −4.963 kcal/moL and −5.039 kcal/moL, respectively, at active sites 1 and 2 (Figure 6a,b). Analysis of the active site revealed that there was a salt bridge between the ligand and the Arg18 residue of the protein, as well as a strong hydrogen bond between the ligand and Phe93.

## 4. Discussion

Noncommunicable diseases, such as cancer, diabetes, and obesity, are undoubtedly the leading causes of mortality in the world [1]. The primary problem with existing medication is that patients experience adverse side effects that may potentially compromise their daily activities [21]. Scientific investigation has now confirmed a possible link between cancer, diabetes, and obesity [48,49]. Therefore, a single medicinal remedy free of adverse effects that can counteract these conditions is the ideal solution [50]. The ability of the plant extract of *N. arbor-tristis* to fulfill this gold standard has been investigated in the present study. 

Phytochemicals are naturally occurring, plant-based chemicals that have been proven to be effective against chronic diseases, such as cancer, diabetes, and even obesity. Therefore, extracts that are potentially efficient against diseases have been shown to be especially rich in polyphenols and flavonoids [51]. Khanapur et al. [20] obtained high flavonoid and phenolic contents in the ethanol crude extract, followed by the ethanol and the butanol fractions. However, in the present study, the crude aqueous extract and the chloroform fraction exhibited a high amount of polyphenols and flavonoids. The difference of the results obtained in the present study can be attributed to different extraction methods employed in our study. Here, we used water extraction of *N. abor-tristis* flowers, whereas Khanapur et al. [20] used ethanol extraction The presence of flavonoids confirm the ability of *N. arbor-tristis* flower extract to potentially treat abnormal cell replication in diseases such as cancer and assist in the regulation of blood glucose levels, thus having the ability to combat obesity [52]. 

Small intestinal α-amylase is a key enzyme of dietary carbohydrate digestion in humans [53]. Once carbohydrates are digested, glucose, the resulting product of carbohydrates, is absorbed into the circulatory system, increasing the postprandial blood glucose level. Therefore, in the present study, it was evident that *N. arbor-tristis* could be used in the management of postprandial glucose increase, since the extract can inhibit the α-amylase enzyme, and increase the glucose transportation across the cells. The present findings confirm that *N. arbor-tristis* exerts its hypoglycemic activity via the inhibition of the α-amylase enzyme and hence, flowers of *N. abor-tristis* can be considered as being beneficial in the management of diabetes and obesity. The results agreed with our previous results observed in mice [21].

According to the results, the hexane, ethyl acetate, and methanol fractions of *N. abor-tristis* exhibited potent α-amylase inhibitory activity compared to the standard drug, acarbose. The potential enzyme inhibitory activity can be attributed to both phenolic and flavonoid compounds observed in the fractions. This is the first report of hypoglycemic activities of solvent fractions from an aqueous extract of the *N. abor-tristis* flowers.

Glucose uptake by the yeast cell membrane has been widely studied to evaluate the hypoglycemic activity of plant compounds. Glucose is the preferred energy source of yeast, and according to the available literature, a variety of glucose-signaling pathways have been characterized. Ronald et al. [54] reported that yeast is an excellent model to study glucose-sensing mechanisms, which occur via facilitated diffusion similar to the mammalian intestinal lumen. Facilitated carriers enable the transportation of solutes down the concentration gradient, and, therefore, glucose is only transported if intracellular glucose is effectively removed [55]. The present data suggest that the aqueous extract and its fractions, particularly the ethyl acetate and hexane fractions, were able to enhance glucose uptake effectively. On the contrary, at all concentrations, particularly at 5 mg/mL, aqueous extract may be capable of enriching efficient utilization to control glucose levels effectively [56]. This also implies that fractions may help to retain the glucose in the intestinal lumen at the concentrations used in the study [57,58].

The inhibition of α-amylase is expected to be a better suppressor of postprandial hyperglycemia, as the inhibitor would not produce glucose via hydrolysis of carbohydrates [59]. Thus, the inhibition of α-amylase could also lead to the impaired uptake of glucose by yeast cells. The assay performed in the present study revealed the therapeutic potential of the ethyl acetate, methanol, and hexane fractions of *N. arbor-tristis* to reduce the postprandial blood glucose level by minimizing glucose uptake and disturbing the α-amylase enzyme.

The diabetic condition is associated with hyperlipidemia [58] and obesity. Excess glucose is converted into tri-glyceraldehyde and deposited in fatty tissues. Thus, alterations in the serum lipid levels are apparent during diabetic conditions, resulting in an increased risk for coronary heart ailments [60]. Diabetic conditions can lead to lipolysis and the mobilization of free fatty acids from adipose tissues [61]. The results indicated that the differentiation of 3T3-L1 cells was observed in the ethyl acetate and chloroform fractions, followed by the hexane fraction, in the presence of the differentiation cocktail. It was observed that the crude extract and the methanol fraction induced the differentiation alone, whereas the other fractions needed the cocktail to induce adipocyte differentiation, indicating that the hexane and ethyl acetate fractions had the ability to inhibit the adipocyte proliferation and differentiation of 3T3-L1 cells. To our knowledge, this is the first report to demonstrate *N. abor-tristis* flower extract inhibiting the differentiation of 3T3-L1 cells. High levels of gatekeeper enzyme lipoprotein lipase have been found in differentiated 3T3-L1 cells [62]. The inhibition of lipoprotein lipase by the *N. abor-tristis* flower extract and its fractions could lead to the impairment of 3T3-L1 cell differentiation. Further, lipoprotein lipase secreted by adipocytes plays an important role in the hydrolysis of plasma triacylglycerols into free fatty acids [63], which will be taken up by adipocytes, thus escalating the formation of adipose tissue. Previously we reported that the aqueous extract of *N. abor-tristis* flowers has the ability to reduce the triglyceride levels in mice blood [21]. The increased uptake of glucose observed with the hexane and ethyl acetate fractions could also lead to a decrease in the differentiation of adipocyte cells [64]. 

The apoptosis assay to determine the cell growth or cell death has been widely used to identify potential anticancer agents. In this study, *N. abor-tristis* and its fractions were tested for their antiproliferative activity using human leukemic primary cells, T lymphoblastic leukemia cells, and a breast cancer cell line. Among the tested fractions and the crude aqueous extract of *N. arbor-tristis*, the methanol fraction and the crude extract demonstrated the highest antiproliferative activity against the Jurkat T cells. The concentration needed to reduce the growth of Jurkat T cells to 50% was 987 and 733 μM, respectively, by the crude extract and the methanol fraction. However, this activity was fairly low compared to the standard drug used.

Anti-AML and anti-CLL efficacy for the crude aqueous extract and the fractions was demonstrated using apoptosis assays. The extract and the fractions (except for the PBMC from AML patients) exhibited potent inhibitory activities on cell proliferation in leukemic cells compared to the control PBMC cells. The control drug, doxorubicin, exhibited the highest inhibitory activities against the primary leukemic cells, as well as other cell lines used in the present study. However, the disadvantage of doxorubicin is that it is toxic to both cancer and normal cells, which is evident by the obtaining the lowest IC_50_ value for control PBMC cells. Hence, to avoid disparities, the selectivity index (SI) was developed by Bartmanska [47]. The result of the present study indicates that the investigated extract and fractions were selective toward the PBMC cells obtained from CLL patients. It should be emphasized that the ethyl acetate and methanol fractions possess compounds with a very high selectivity and specificity action. Similarly, the ethyl acetate, chloroform, and methanol fractions can also be considered as containing compounds with a high selectivity against AML cells. Hence, if the aqueous extract of *N. abor-tristis* flowers can be developed as a nutraceutical, it can effectively prevent leukemia when taken as a supplement in combination with a known anticancer drug. The results also emphasized that care should be taken when prescribing doxorubicin as an anticancer drug due to its nonspecific action against all the cells including normal cells. 

Khanpur et al. [20] reported the antiproliferative activity of the ethanol and ethyl acetate extracts of *N. abor-tristis* flowers against the MCF7 breast cancer cell line. However, in the present study, we describe that the hexane fraction, followed by the methanol and ethyl acetate fractions of the aqueous extract, exhibited inhibitory activity against the breast cancer cell line. However, because we did not use a control breast cell line, we could not calculate the specificity index. In South Asian countries, women are frequently identified with estrogen-dependent cancers, and the MCF7 line is an estrogen-dependent cell line. Hence, further studies with the normal cell line will enable the development of anticancer drugs with high specificity. The data also suggest that the aqueous extract of *N. arbor-tristis* flowers and its fractions have antiproliferative activity on human cancer cells and may have potential beneficial effects against chronic lymphocytic leukemia.

The GC/MS analysis of the chloroform fraction of *Nyctanthes arbor-tristis* flowers revealed the presence of 4-hydroxypyridine-1-oxide [65], 2-thiophenecarbonyl chloride [34], and phenol,2,5-bis(1,1-dimethylethyl) [35] with strong anticancer activity. Among the compounds identified in the hexane fraction, 1-octadecene [37] and 1-heptacosanol [46] exhibited strong anticancer properties. Though eight compounds were recorded in the ethyl acetate fraction, only three compounds showed recorded biological activities, and none of them reported anticancer activity. Hexanedioic acid, bis(2-ethylhexyl) ester, and 2,5-dimethylhexane-2,5-dihydroperoxide in the ethyl acetate fraction and 2-chloropropionic acid, octadecyl ester, 1-heptacosanol, and 1-octadecene in the hexane fraction showed strong antioxidant activity. Oxidative stress plays a major role in various diseases via cell damage and malfunction. Excessive levels of blood lipid and glucose and cell proliferation can induce elevated oxidative stress both in vitro and in vivo [66]. Hence, these bioactive compounds have the ability to scavenge reactive oxygen species, reducing adipocyte differentiation, cell proliferation, and blood glucose concentration.

Survivin is a member of the inhibitor of apoptosis (IAP) protein family that inhibits caspases, leading to cancer cell survival. Being a protooncogene, survivin plays a critical role in the regulation of mitosis by inhibiting apoptosis. In the case of a mutation, this could potentially become an oncogene, which is highly expressed in most cancers, leading to a poor clinical outcome [67]. According to in silico analysis, in both phenol,2,5-bis(1,1-dimethylethyl) and 4-hydroxypyridine 1-oxide, two potential active sites (for each compound) were identified. With phenol,2,5-bis(1,1-dimethylethyl), both active sites formed a complex with survivin protein, with gliding scores of –5.449 kcal/moL and −5.269 kcal/moL. The 2-dimential analysis of the active sites showed the involvement of one strong hydrogen bond with Lys91 residue and a number of van der Waals forces between the ligand and residues, which formed a complex with the protein. Similarly, with 4-hydroxypyridine 1-oxide, both active sites formed complexes with survivin, with gliding scores of −4.963 kcal/moL and −5.039 kcal/moL. Two-dimensional analysis of the active sites revealed that there was a salt bridge between the ligand and the Arg18 residue of the protein, as well as a strong hydrogen bond between the ligand and Phe93. The study conducted by Quispe et al. [68] displayed a binding site in survivin that is capable of inducing an allosteric conformational change, effectively disrupting the dimer form of survivin and inducing apoptosis. The study goes into detail by displaying some of the residues (Trp10, Phe93, Leu98, Phe101, Glu94, Leu96) in the active site that interact with the potential molecules that induce apoptosis. Hence, it is highly evident that the formation of a ligand–protein complex by both phytochemicals could induce apoptosis by inducing a conformational change to survivin protein. 

In summary, the preliminary screening study on the aqueous extract of *Nyctanthes abor-tristis* flowers and its hexane and ethyl acetate fractions were found to be a potent herbal approach for the management of postprandial glucose increase and obesity by inhibiting lipid and carbohydrate metabolizing enzymes compared to other plants. Similarly, the ethyl acetate and methanol fractions were found to be specifically beneficial to develop as a neutrical against acute lymphatic and chronic myeloid leukemia cancers. In silico analysis showed that both phenol,2,5-bis(1,1-dimethylethyl) and 4-hydroxypyridine 1-oxide possess anticancer properties. Hence, our study provides a new arena for the treatment of obesity and leukemia.

## Figures and Tables

**Table 1:** Total phenolic and flavonoid contents of the crude aqueous extract and different fractions of *Nyctanthes abor-tristis* flowers.

Aqueous Extract and Its Fractions	Total Phenolic Content	Total Flavonoid Content
	mg GAE/g	mg QE/g
Aqueous	160 ± 0.06	600 ± 1.25
Chloroform	351 ± 0.0004	120 ± 0.93
Hexane	180 ± 0.0003	24 ± 0.06
Ethyl acetate	285 ± 0.001	30 ± 0.03
Methanol	450 ± 0.014	528 ± 2.01

The values are presented as the mean ± standard error mean (*n* = 3). GAE: gallic acid, QE: Quercetin.

**Table 2:** Active compounds identified in the chloroform fraction of *Nyctanthes arbor-tristis* aqueous flower extract by as chromatography/mass spectrometry (GC/MS) analysis.

Retention Time	Abundance (%)	Name	Molecular Formula	Compound Class	Reported Activity
9.094	4.261	4-hydroxypyridine-1-oxide	C_5_H_5_NO_2_	Pyridine derivative	Anticancer [32]
9.307	4.055	2-aminopyrimidine-1-oxide	C_4_H_4_BrN_3_O	Heterocyclic	Antimicrobial [33]
9.387	3.430	2-thiophenecarbonyl chloride	C_5_H_3_ClOS	*Hydrazonoyl chloride*	Anticancer [34]
9.463	7.199	5-ethylcyclopent-1-ene-1-carboxylic acid	C_8_H_12_O_2_	Organic acid	Not reported
12.034	17.849	phenol, 2,5-bis(1,1-dimethyl ethyl)	C_14_H_22_O	Phenolic compound	Anticancer [35]
12.953	5.836	7-hexadecene	C_16_H_32_	Alkene	Antioxidant, antimicrobial [36]
15.172	8.088	1-octadecene	C_18_H_36_	Alkene	Antibacterial, antioxidant, anticancer [37]
17.182	8.372	1-eicosene	C_20_H_40_	Alkene	Antimicrobial [38], anticancer [39]
18.636	3.091	hentriacontane	C_31_H_64_	Alkane	Anti-inflammatory [40], anticancer [41]
20.703	6.164	1-docosene	C_29_H_60_	Alkene	Antibacterial [37], anticancer [42]

**Table 3:** Active compounds identified in the ethyl acetate fraction of *Nyctanthes arbor-tristis* aqueous flower extract by gas chromatography/mass spectrometry (GC/MS) analysis.

Retention Time	Abundance (%)	Compound Name	Molecular Formula	Compound Class	Reported Activity
7.656	11.532	cyclopentanecarboxamide, *N*-(2-fluorophenyl)	C_12_H_14_FNO	Heterocyclic compound	Not reported
7.910	1.918	2-thiophenecarboxylic acid, 3,5- dimethylcyclohexyl ester	C_13_H_18_O_2_S	Organic acid	Not reported
8.314	13.592	2,5-dimethylhexane-2,5-dihydroperoxide	C_8_H_18_O_4_	Organic compound	Anti-inflammatory, antioxidant [43]
8.467	8.925	adipic acid-ethyl propargyl ester	C_11_H_16_O_4_	Organic acid	Not reported
9.342	7.337	cyclopentanecarboxamide, *N*-(2-fluorophenyl)	C_12_H_14_FNO	Heterocyclic compound	Not reported
9.435	32.655	succinic acid (3,5-dimethylcyclohexy) ester	C_20_H_34_O_4_	Organic acid	Not reported
13.162	3.071	diethyl phthalate	C_12_H_14_O_4_	Diester of phthalic acid	Antimicrobial [44]
20.721	7.337	hexanedioic acid, bis (2-ethylhexyl) ester	C_22_H_42_O_4_	Organic acid	Antioxidant [45]

**Table 4:** Active compounds identified in the hexane fraction of *Nyctanthes arbor-tristis* aqueous flower extract by gas chromatography/mass spectrometry (GC/MS) analysis.

Retention Time	Abundance (%)	Name	Molecular Formula	Compound Class	Reported Activity
13.345	4.950	2-chloropropionic acid, octadecyl ester	C_21_H_41_ClO_2_	Organic acid	Antimicrobial, antioxidant [44]
15.343	25.685	1-octadecene	C_18_H_36_	Alkene	Antibacterial, antioxidant, anticancer [37]
17.271	17.759	pentafluoropropionic acid, hexadecyl ester	C_19_H_33_F_5_O_2_	Organic acid	Not reported
19.075	14.333	1-heptacosanol	C_27_H_56_O	Fatty alcohol	Antimicrobial, antioxidant, anticancer [46]
20.754	7.135	pentafluoropropionic acid, hexadecyl ester	C_19_H_33_F_5_O_2_	Organic acid	Not reported

**Table 5:** IC_50_ values exhibited by *N. abor-tristis* aqueous extract and its fractions against the inhibitory activity of the enzyme α-amylase.

Extract/Fraction	IC_50_ (mg/mL)
Crude aqueous extract	2.223 ± 0.02
Chloroform fraction	12.68 ± 0.09
Hexane fraction	0.665 ± 0.01 **
Ethyl acetate fraction	0.718 ± 0.01 **
Methanol fraction	1.504 ± 0.34
Acarbose (standard)	2.45 ± 0.23

The values are presented as the mean ± SEM (*n* = 3). ** *p* < 0.01.

**Table 6:** IC_50_ values exhibited by the crude aqueous extract of *N. abor-tristis* flowers and its four fractions against the inhibitory activity on primary peripheral blood mononuclear cells (PBMC) from healthy adults (control PBMC), primary peripheral blood mononuclear cells from acute myeloid leukemia patients (PBMC-AML), and primary peripheral blood mononuclear cells from chronic lymphocytic leukemia (PBMC-CLL) patients, the MFC7 breast cancer cell line, and human leukemic T cell lymphoblasts (Jurkat cells).

**IC_50_ (Inhibition of killing) µg/mL**	**Extract/Fraction**	**Jurkat Cells**	**MCF7**	**PBMC-AML**	**PBMC-CLL**	**Control PBMC**
	Crude aqueous	987	1664	1262	90.1	1588
	Methanol	733	1016	1986	138	6654
	Ethyl acetate	70500	1493	2994	60.3	6179
	Hexane	1174	717.6	9710	273	2520
	Chloroform	1394	38,210	6169	98.6	8894
	Doxorubicin (standard)	8.19 × 10^−2^	4.62	3.39	6.08 × 10^−2^	9.00 × 10^−2^

**Table 7:** Selectivity indices of tested crude aqueous extract, its fractions, and the standard drug.

Extract/Fraction	PBMC-AML (SI_A_)	PBMC-CLL (SI_B_)
Crude aqueous extract	1.26	17.6
Methanol fraction	3.35	48.22
Ethyl acetate fraction	2.06	102.5
Hexane fraction	0.26	9.23
Chloroform fraction	1.44	98.20
Doxorubicin (standard drug)	0.03	1.5

The selectivity index (SI) was calculated for each compound using the following formula: SI_A_ = IC_50_ cells from healthy patients (PMBC)/IC_50_ PMBC-AML cancerous cells; SI_B_ = IC_50_ cells of healthy patients (PMBC)/IC_50_ for CLL cancerous cells. SI < 1.0 is a nonselective action. PBMC: primary peripheral blood mononuclear cells; AML: adult acute myeloid; CLL: chronic lymphocytic leukemia primary cells.
